# Supplementary material for: MetaQTL: a package of new computational methods for the meta-analysis of QTL mapping experiments
Source: BMC Bioinformatics. 2007 Feb 8;8:49. doi: 10.1186/1471-2105-8-49 (PMC1808479; doi:10.1186/1471-2105-8-49)
Supplement: Additional File 7 — MetaQTL Package : jar file and tutorial. This Zip archive contains both the MetaQTL JAR file and the files of the tutorial. [file 1471-2105-8-49-S7.zip › org.inra.metaqtl/doc/index-files/index-3.html]

C-Index


|  |  |  |
| --- | --- | --- |
| **Overview** \| Package \| Class \| Use \| **Tree** \| **Deprecated** \| **Index** \| **Help** | |  |
| **PREV LETTER**   **NEXT LETTER** | **FRAMES**    **NO FRAMES**     **All Classes** |  |


A B C D E F G H I K L M N O P Q R S T U V W X Y Z 

---


## **C**

**C** - Variable in class org.metaqtl.util.OffspringManager.HaldaneWaddingtonProportion: **c1** - Variable in class org.metaqtl.Qtl: The recombination rate between the QTL position and the left flanking marker. **c2** - Variable in class org.metaqtl.Qtl: The recombination rate between the QTL position and the right flanking marker. **C\_coeff** - Static variable in class org.metaqtl.util.OffspringManager.HaldaneWaddingtonProportion: **canAddMoreQtl(double)** - Method in class org.metaqtl.graph.QtlLayerBuffer: Retruns true if more Qtl can be added to the configuration, false otherwise. **card** - Variable in class org.metaqtl.TreeNode: The number of sequences in the cluster **cartoFunction** - Static variable in interface org.thalia.bio.entity.IGeneticMapProperties: **cartoFunctionHaldane** - Static variable in interface org.thalia.bio.entity.IGeneticMapProperties: **cartoFunctionKey** - Static variable in interface org.thalia.bio.entity.IGeneticMapProperties: **cartoFunctionKosambi** - Static variable in interface org.thalia.bio.entity.IGeneticMapProperties: **ccov** - Variable in class org.metaqtl.EMResult: The complete information (just for means). **CENTROID\_METHOD** - Static variable in class org.metaqtl.algo.HClustAlgorithm: Centroid method **CentroidMethod(double[], double[])** - Static method in class org.metaqtl.algo.HClustAlgorithm: This method performs a hierarchical clustering of the given data points using a centroid method based on the mahalanobis distance. **cg** - Variable in class org.metaqtl.Chromosome: The number of generations of the offspring population. **checkProjection(ChromCluster[])** - Method in class org.metaqtl.algo.QtlProjAlgorithm: **chi2** - Variable in class org.metaqtl.MetaChrom: The chi-square value of this chromosome. **chi2** - Variable in class org.metaqtl.MultiFitResult: The chi-square of the model. **children** - Variable in class org.metaqtl.TreeNode: Left and right children, or null **children** - Variable in class org.thalia.bio.entity.bean.OntologyTermBean: The children of the term. **CHISQUARE** - Static variable in interface org.metaqtl.IBioLGroupProperties: The key for the chisquare value. **chrom** - Variable in class org.metaqtl.ChromCluster.ClusterEdge: **CHROM\_ALIGN\_MODE** - Static variable in class org.metaqtl.graph.MetaGraphPar: **CHROM\_DISTANCE\_SCALE** - Static variable in class org.metaqtl.graph.MetaGraphPar: **CHROM\_FLANKING\_CEX** - Static variable in class org.metaqtl.graph.MetaGraphPar: The default cex parameter for flanking regions at the top and the end of the chromosome. **CHROM\_NAME\_FONT** - Static variable in class org.metaqtl.graph.MetaGraphPar: **CHROM\_NAME\_HSPACE** - Static variable in class org.metaqtl.graph.MetaGraphPar: **CHROM\_STROKE** - Static variable in class org.metaqtl.graph.MetaGraphPar: The stroke of the chromosome shape. **CHROM\_STROKE\_COLOR** - Static variable in class org.metaqtl.graph.MetaGraphPar: The color of the stroke of the Chromosome shape. **CHROM\_TICK\_COLOR** - Static variable in class org.metaqtl.graph.MetaGraphPar: The color of the marker ticks on the chromosome. **CHROM\_TICK\_STROKE** - Static variable in class org.metaqtl.graph.MetaGraphPar: **CHROM\_TICK\_WIDTH\_1** - Static variable in class org.metaqtl.graph.MetaGraphPar: The width of the marker ticks. **CHROM\_TICK\_WIDTH\_2** - Static variable in class org.metaqtl.graph.MetaGraphPar: **CHROM\_TICK\_WIDTH\_3** - Static variable in class org.metaqtl.graph.MetaGraphPar: **CHROM\_WIDTH** - Static variable in class org.metaqtl.graph.MetaGraphPar: The width of the chromosome. **ChromAxe** - Class in org.metaqtl.graph: **ChromAxe(double, double, double)** - Constructor for class org.metaqtl.graph.ChromAxe: **ChromCluster** - Class in org.metaqtl: A cluster of chromosomes which share the same name inside a `MapCluster` framwork. **ChromCluster()** - Constructor for class org.metaqtl.ChromCluster: Creates a new instance if ChromCluster. **ChromCluster.ClusterEdge** - Class in org.metaqtl: **ChromCluster.ClusterEdge(CMarkerSequence, Chromosome)** - Constructor for class org.metaqtl.ChromCluster.ClusterEdge: **ChromCluster.ClusterGraph** - Class in org.metaqtl: **ChromCluster.ClusterGraph(ChromCluster)** - Constructor for class org.metaqtl.ChromCluster.ClusterGraph: **ChromGraph** - Class in org.metaqtl.graph: A `ChromGraph` allows to link together a `Chromosome` and a `QtlPartition` in order to be depicted into a graphic. **ChromGraph()** - Constructor for class org.metaqtl.graph.ChromGraph: **ChromLayer** - Class in org.metaqtl.graph: **ChromLayer(double, double)** - Constructor for class org.metaqtl.graph.ChromLayer: **ChromLayer.CMrkTickPath** - Class in org.metaqtl.graph: **ChromLayer.CMrkTickPath()** - Constructor for class org.metaqtl.graph.ChromLayer.CMrkTickPath: **ChromLayer.CMrkTickPoint** - Class in org.metaqtl.graph: **ChromLayer.CMrkTickPoint()** - Constructor for class org.metaqtl.graph.ChromLayer.CMrkTickPoint: **chromNames** - Variable in class org.metaqtl.MetaQtlAnalysis: The names of the chromosomes. **chromNames** - Variable in class org.metaqtl.MetaQtlModel: The chromosomes. **Chromosome** - Class in org.metaqtl: On a Chromosome there are two types of locus : Marker QTL (Quantitative Trait Loci) inside a mapping context which is defined by both the mapping framework (function and unit) and the population cross design used to build this chromosome. **Chromosome()** - Constructor for class org.metaqtl.Chromosome: **chromosome** - Variable in class org.metaqtl.graph.ChromGraph: The chromosome to draw **ChromosomeAdapter** - Class in org.metaqtl.adapter: This class defines some usefull methods to adapt `Chromosome` objects into other kinds of objects or the inverse. **ChromosomeAdapter()** - Constructor for class org.metaqtl.adapter.ChromosomeAdapter: **chromosomes** - Variable in class org.metaqtl.MetaMap: The meta chromosomes. **ChromShape** - Class in org.metaqtl.graph: **ChromShape()** - Constructor for class org.metaqtl.graph.ChromShape: **ci** - Variable in class org.metaqtl.Qtl: The QTL confidence interval. **ci2sd(double)** - Static method in class org.metaqtl.numrec.NumericalUtilities: **ci\_height** - Variable in class org.metaqtl.graph.QtlShape: **CI\_MISS\_IGNORE** - Static variable in interface org.metaqtl.IMetaQtlConstants: MSD Mode : ignore QTL with missing standard deviation. **CI\_MISS\_IMPUTATION** - Static variable in interface org.metaqtl.IMetaQtlConstants: MSD Mode : replace missing standard deviation by the mean of the observed ones. **CI\_MODE\_AVAILABLE** - Static variable in interface org.metaqtl.IMetaQtlConstants: SD Mode : use the available information to compute the standard deviation. **CI\_MODE\_LOD** - Static variable in interface org.metaqtl.IMetaQtlConstants: SD Mode : use only the confidence interval information if defined to compute the standard deviation. **CI\_MODE\_MAXIMAL** - Static variable in interface org.metaqtl.IMetaQtlConstants: SD Mode : compute the standard deviation using both lod support and rsquare information (if available) and then choose the maximal standard-deviation. **CI\_MODE\_RSQUARE** - Static variable in interface org.metaqtl.IMetaQtlConstants: SD Mode : use only the rsquare information if defined to compute the standard deviation. **CI\_WIDTH** - Static variable in class org.metaqtl.graph.QtlLayer: The default width of the CI of the Qtl **ci\_width** - Variable in class org.metaqtl.graph.QtlShape: **clog** - Variable in class org.metaqtl.EMResult: The complete loglikelihood. **ClustAlgorithm** - Class in org.metaqtl.algo: **ClustAlgorithm(IBioGenome, IBioOntology)** - Constructor for class org.metaqtl.algo.ClustAlgorithm: **ClustAlgorithm(IBioGenome, String, IBioOntology)** - Constructor for class org.metaqtl.algo.ClustAlgorithm: **cluster** - Variable in class org.metaqtl.ChromCluster.ClusterGraph: **clusterings** - Variable in class org.metaqtl.MetaQtlResult: The meta qtl clusterings. **clusters** - Variable in class org.metaqtl.ChromCluster: The list of the cluster members. **CMarkerSequence** - Class in org.metaqtl: A Common marker sequence between two ordered set of markers is a sub-set of at least two markers which order is the same between the two sets (may be reverse). **CMarkerSequence(int[][], int)** - Constructor for class org.metaqtl.CMarkerSequence: Initiates a new Common marker sequence from the tow arrays of common marker indices `mcidx[0..1][0..nmc-1]`. **CmdLineParser** - Class in org.metaqtl.main: Largely GNU-compatible command-line options parser. **CmdLineParser()** - Constructor for class org.metaqtl.main.CmdLineParser: **CmdLineParser.IllegalOptionValueException** - Exception in org.metaqtl.main: Thrown when an illegal or missing value is given by the user for an option that takes a value. **CmdLineParser.IllegalOptionValueException(CmdLineParser.Option, String)** - Constructor for exception org.metaqtl.main.CmdLineParser.IllegalOptionValueException: **CmdLineParser.NotFlagException** - Exception in org.metaqtl.main: Thrown when the parsed commandline contains multiple concatenated short options, such as -abcd, where one or more requires a value. **CmdLineParser.Option** - Class in org.metaqtl.main: Representation of a command-line option **CmdLineParser.Option.BooleanOption** - Class in org.metaqtl.main: **CmdLineParser.Option.BooleanOption(char, String)** - Constructor for class org.metaqtl.main.CmdLineParser.Option.BooleanOption: **CmdLineParser.Option.BooleanOption(String)** - Constructor for class org.metaqtl.main.CmdLineParser.Option.BooleanOption: **CmdLineParser.Option.DoubleOption** - Class in org.metaqtl.main: An option that expects a floating-point value **CmdLineParser.Option.DoubleOption(char, String)** - Constructor for class org.metaqtl.main.CmdLineParser.Option.DoubleOption: **CmdLineParser.Option.DoubleOption(String)** - Constructor for class org.metaqtl.main.CmdLineParser.Option.DoubleOption: **CmdLineParser.Option.IntegerOption** - Class in org.metaqtl.main: An option that expects an integer value **CmdLineParser.Option.IntegerOption(char, String)** - Constructor for class org.metaqtl.main.CmdLineParser.Option.IntegerOption: **CmdLineParser.Option.IntegerOption(String)** - Constructor for class org.metaqtl.main.CmdLineParser.Option.IntegerOption: **CmdLineParser.Option.LongOption** - Class in org.metaqtl.main: An option that expects a long integer value **CmdLineParser.Option.LongOption(char, String)** - Constructor for class org.metaqtl.main.CmdLineParser.Option.LongOption: **CmdLineParser.Option.LongOption(String)** - Constructor for class org.metaqtl.main.CmdLineParser.Option.LongOption: **CmdLineParser.Option.StringOption** - Class in org.metaqtl.main: An option that expects a string value **CmdLineParser.Option.StringOption(char, String)** - Constructor for class org.metaqtl.main.CmdLineParser.Option.StringOption: **CmdLineParser.Option.StringOption(String)** - Constructor for class org.metaqtl.main.CmdLineParser.Option.StringOption: **CmdLineParser.OptionException** - Exception in org.metaqtl.main: Base class for exceptions that may be thrown when options are parsed **CmdLineParser.UnknownOptionException** - Exception in org.metaqtl.main: Thrown when the parsed command-line contains an option that is not recognised. **CmdLineParser.UnknownSuboptionException** - Exception in org.metaqtl.main: Thrown when the parsed commandline contains multiple concatenated short options, such as -abcd, where one is unknown. **cms** - Variable in class org.metaqtl.ChromCluster.ClusterEdge: **CMSAlgorithm** - Class in org.metaqtl.algo: Use this class to find common marker sequences between two chromosomes. **CMSAlgorithm()** - Constructor for class org.metaqtl.algo.CMSAlgorithm: **COLLECTION** - Static variable in interface org.thalia.bio.IBioEntityTypes: **color2string(Color)** - Static method in class org.metaqtl.graph.MetaGraphPar: **COMMON\_STROKE\_WIDTH** - Static variable in class org.metaqtl.graph.MetaGraphPar: **compare(Object, Object)** - Method in class org.thalia.bio.util.BioEntityNameComparator: **compare(Object, Object)** - Method in class org.thalia.bio.util.LocusPositionComparator: **computeCOV(double[], EMResult)** - Static method in class org.metaqtl.algo.EMAlgorithm: Computes the variance-covariance matrix of the mixture component estimates. **computeCriteria()** - Method in class org.metaqtl.EMResult: This methos computes the following criteria : AIC AIC3 ICOMP MIR BIC AWE **computeDM(double[], double[], double[], EMResult)** - Static method in class org.metaqtl.algo.EMAlgorithm: Computes the matrix of derivatives of the update function. **computeQtlSD(int)** - Method in class org.metaqtl.Chromosome: According to the given standard deviation computation mode this method computes for each QTL the value of the standard deviation of the QTL position. **ConsMap** - Class in org.metaqtl.main: **ConsMap()** - Constructor for class org.metaqtl.main.ConsMap: **copy(EMResult, EMResult)** - Static method in class org.metaqtl.EMResult: **cov** - Variable in class org.metaqtl.MultiFitResult: The variance-covariance matrix of the parameter estimates. **criteria** - Variable in class org.metaqtl.EMResult: The model choice criteria. **cross** - Variable in class org.metaqtl.Qtl: The QTL cross design. **CROSS\_SIZE** - Static variable in interface org.metaqtl.IBioGenomeProperties: The cross size key. **CROSS\_TYPE** - Static variable in interface org.metaqtl.IBioGenomeProperties: The cross type key. **crossLayers** - Variable in class org.metaqtl.graph.MetaGraph: **CrossPopulation** - Class in org.thalia.bio.entity: Class Description Here **CrossPopulation()** - Constructor for class org.thalia.bio.entity.CrossPopulation: **CrossPopulation(String, IBioEntity)** - Constructor for class org.thalia.bio.entity.CrossPopulation: **CrossPopulation(String, IBioEntity, int)** - Constructor for class org.thalia.bio.entity.CrossPopulation: **crossToString(int, int)** - Static method in class org.thalia.bio.util.BioCrossTypes: **cs** - Variable in class org.metaqtl.Chromosome: The size of the offspring population. **css** - Variable in class org.metaqtl.CMarkerSequence: The sizes of the common sequences **ct** - Variable in class org.metaqtl.Chromosome: The type of the offspring population.

---


|  |  |  |
| --- | --- | --- |
| **Overview** \| Package \| Class \| Use \| **Tree** \| **Deprecated** \| **Index** \| **Help** | |  |
| **PREV LETTER**   **NEXT LETTER** | **FRAMES**    **NO FRAMES**     **All Classes** |  |


A B C D E F G H I K L M N O P Q R S T U V W X Y Z 

---
